# *N*^6^-methyladenosine-dependent pri-miR-17-92 maturation suppresses PTEN/TMEM127 and promotes sensitivity to everolimus in gastric cancer

**DOI:** 10.1038/s41419-020-03049-w

**Published:** 2020-10-09

**Authors:** Yiting Sun, Song Li, Wenbin Yu, Zeyi Zhao, Jing Gao, Cheng Chen, Meng Wei, Teng Liu, Lanbo Li, Lian Liu

**Affiliations:** 1 Department of Medical Oncology, Qilu Hospital, Cheeloo College of Medicine, Shandong University, Jinan, Shandong 250012 China; 2 Department of Medical Oncology, National Cancer Center/National Clinical Research Center for Cancer/Cancer Hospital, Chinese Academy of Medical Sciences & Peking Union Medical College, Beijing, 100021 China; 3 Department of General Surgery, Qilu Hospital, Cheeloo College of Medicine, Shandong University, Jinan, Shandong 250012 China; 4 Animal Laboratory, Qilu Hospital, Cheeloo College of Medicine, Shandong University, Jinan, Shandong 250012 China

**Keywords:** Gastric cancer, Genetics research

## Abstract

*N*^6^-methyladenosine (m^6^A) is the most common epigenetic RNA modification with essential roles in cancer progression. However, roles of m^6^A and its regulator METTL3 on non-coding RNA in gastric cancer are unknown. In this study, we found elevated levels of m^6^A and METTL3 in gastric cancer. Increased METTL3 expression indicated poor outcomes of patients and high malignancy in vitro and in vivo. Mechanically, m^6^A facilitated processing of pri-miR-17-92 into the miR-17-92 cluster through an m^6^A/DGCR8-dependent mechanism. The m^6^A modification that mediated this process occurred on the A879 locus of pri-miR-17-92. The miR-17-92 cluster activated the AKT/mTOR pathway by targeting *PTEN* or *TMEM127*. Compared with those with low levels of METTL3, METTL3-high tumors showed preferred sensitivity to an mTOR inhibitor, everolimus. These results reveal a perspective on epigenetic regulations of non-coding RNA in gastric cancer progression and provide a theoretical rationale for use of everolimus in the treatment of m^6^A/METTL3-high gastric cancer.

## Introduction

Gastric cancer is one of the most common malignancies and the third leading cause of cancer-related death worldwide^1^. With few specific symptoms in early stages and with low rates of gastroscopy, most patients have already reached an advanced stage at the time of initial diagnosis. Even among patients who underwent curative resection, 60% suffered recurrences and distant metastasis, with a median overall survival (mOS) of <12 months^2^. Moreover, gastric cancers with peritoneal metastasis respond rarely to any treatments, leading to an extremely inferior prognosis with life expectancy <6 months^3^. The effect of targeted therapy is quite limited by the lack of dominant driver genes in gastric cancer. Trastuzumab is the only target drug approved for the first-line treatment of advanced gastric cancer based on the TOGA trial, but its usage was confined in a small part of the patients with ERBB2 amplification^4^. The anti-angiogenic drug bevacizumab only improves overall survival in non-Asian patients as the first-line treatment^5^. Up to now, the immune checkpoint inhibitors, pembrolizumab (KEYNOTE-059 cohort 1)^6^ and nivolumab (ATTRACTION-02)^7^, are only approved for third-line and later treatment in gastric cancer, with response rates <15%. Therefore, an in-depth investigation of the molecular mechanisms in gastric cancer oncogenesis and progression is critical to allow early diagnosis, innovative therapeutic methods, and ultimately improved prognosis and quality of life for patients.

*N*^6^-Methyladenosine (m^6^A) is the most common epigenetic modification in eukaryotic messenger RNA (mRNA)^8^ and non-coding RNA (ncRNA)^9^. It plays a crucial role in gene expression by participating in almost every stage of mRNA metabolism and exerts vital and specific roles in the pathogenesis of various cancers^10^. Recently, several studies reported that METTL3, the core methyltransferase for m^6^A modification, promotes gastric cancer progression^11,12^. However, these studies only focused on m^6^A of mRNA, and few studies investigated m^6^A of ncRNA in gastric cancer or implied the possible clinical translational value of m^6^A/METTL3 related signaling pathways.

Here, we investigated the biological function of METTL3/m^6^A in regulating ncRNA and defined a novel pathway for m^6^A-dependent primary microRNA (miRNA) maturation and AKT/mTOR activation in gastric cancer, which could be counteracted by everolimus.

## Materials and methods

### Cell culture and transfection

AGS, HGC-27, and MKN-45 cell lines were obtained from the Cell Bank, Chinese Academy of Science (Shanghai, China). All cells were authenticated and tested for mycoplasma contamination. Overexpressing plasmids were transfected by Lipofectamine 2000 (Thermo Fisher Scientific, Waltham, MA). Lentivirus containing METTL3 and shRNA against METTL3 (shM3: CCGGCGTCAGTATCTTGGGCAAGTTCTCGAGAACTTGCCCAAGATACTGACGTTTTTG and shM3-2: CCGGGCTGCACTTCAGACGAATTATCTCGAGATAATTCGTCTGAAGTGCAGCTTTTTG) were used for stable transfection, followed by puromycin selection. The miniMIR17HG plasmid was constructed by inserting the 961–1917th nucleotides of the miR-17-92a-1 RNA (GenBank #: NR_027350.1) into the GV146 vector.

### Clinical samples

Fresh tissues were obtained from gastric cancer patients who underwent radical resections at Qilu Hospital of Shandong University between January 2017 and August 2018. Pathologically confirmed gastric cancer paraffin-embedded tissues between 2009 and 2014 were obtained from the Department of Pathology, Qilu Hospital of Shandong University. Only patients with evidence of survival and recurrence were included for OS and RFS analysis, respectively.

### RNA m^6^A quantification

Total RNA was isolated from 10 mm^3^ of fresh tissue or 10^6^ cells using TRIzol (Invitrogen, CA, USA). The m^6^A RNA Methylation Quantification Kit (Abcam, Cambridge, UK) was used to quantify the m^6^A content according to the manufacturer’s instructions. The optical absorbance was measured by a SpectraMax Plus384 Microplate Spectrophotometer (Molecular Device, Sunnyvale, CA, USA).

### Immunohistochemistry (IHC)

In clinical studies, paraffin-embedded sections were blocked by goat serum and stained with anti-METTL3 antibody (Abcam, Cambridge, UK) using an IHC staining kit (Zsbio, Beijing, China). Cell nuclei were stained with hematoxylin. In animal studies, tumor xenografts or peritoneal tumors were fixed and processed with a similar procedure with anti-METTL3, anti-Ki67 (Abcam, Cambridge, UK), and anti-PTEN antibody (CST, Danvers, MA).

### Proliferation and colony-formation assays

For proliferation assays, cells were seeded in 6-well plates (10,000 or 20,000 cells per well) and counted every 24 h. For colony-formation assays, suspended single cells were seeded in 6-well plates (1000 cells per well), and colonies were counted within 14 days.

### Wound-healing, migration, and invasion assays

For wound-healing assays, wounds were made by scratching a line using a 200 µL tip, and the intervals were measured within 72 h. For migration and invasion assays, a Transwell system (Corning, NY, USA) was used as previously described^13^. Migrated and invaded cells were stained with crystal violet (Beyotime, Shanghai, China) and photographed.

### Subcutaneous xenograft and peritoneal implant models

Six-week female BALB/c Nude Mice were purchased from Vital River Laboratory (Beijing, China). For subcutaneous xenograft models, 0.1 mL of cell suspension containing 10^6^ cells were injected subcutaneously into the right flank of mice (*n* = 6 for each group). Mice were sacrificed at 21- or 32-day after injection. For peritoneal implant models, a cell suspension (5 × 10^6^ cells) was injected intraperitoneally. All mice were sacrificed 4 weeks after injection (*n* = 4 for each group). Mice were randomly allocated into each group with no blinding. The animal studies were performed following the ARRIVE guidelines and were approved by the Animal Ethical Committee of Qilu Hospital of Shandong University.

### Western blot

Approximately 25 µg of protein was separated by 10% SDS-PAGE, transferred to 0.22 μm polyvinylidene difluoride membranes (Thermo Fisher Scientific), and probed by primary antibodies. The anti-METTL3, anti-TMEM127, and anti-GAPDH antibodies were purchased from Abcam (Cambridge, UK). The anti-PTEN, anti-phospho-S6K, anti-phospho-S6, and anti-phospho-4E-BP1 antibodies were purchased from Cell Signaling Technology (Danvers, MA, USA).

### RNA immunoprecipitation (RIP)

A Magna RIP RNA-Binding Protein Immunoprecipitation Kit (Millipore, Darmstadt, Germany) was used for RIP. Briefly, cells were lysed and mixed with anti-m^6^A (Abcam, Cambridge, UK), anti-DGCR8 (Abcam, Cambridge, UK) antibodies, or isotype controls (Abcam, Cambridge, UK). The antibody-binding RNA was pulled down by protein A/G magnetic beads and quantified by real-time PCR.

### Quantitative RT-PCR

Quantification of miRNA was performed using an All-in-One™ miRNA qRT-PCR Detection Kit (GeneCopoeia, San Diego, CA, USA). Quantification of primary miRNA and mRNA was performed using an All-in-One™ First-Strand cDNA Synthesis Kit and an All-in-One qPCR Kit (GeneCopoeia, San Diego, CA, USA). Commercial primers for quantitation of miR-17, miR-18, miR-19a, miR-19b-1, miR-20a, miR-92a-1, snRNA U6, METTL3, TMEM127, PTEN, and GAPDH were purchased from GeneCopoeia (San Diego, CA, USA). Primers for pri-miR-17-92 included 5′-CATCTACTGCCCTAAGTGCTCCTT and 5′-GCTTGGCTTGAATTATTGGATGA. Primers for 5S RNA were 5′-TCTCGTCTGATCTCGGAAGC and 5′-AGCCTACAGCACCCGGTATT.

### Everolimus-sensitivity assays

For the in vitro study, everolimus (APExBIO, Houston, TX, USA) was added to cells with final concentrations of 5 or 50 µg/mL. Cell viability was measured using a CCK-8 kit (BestBio, Shanghai, China). For the in vivo study, subcutaneous xenograft models with METTL3-high (*n* = 3) and control cells (*n* = 3) were established as above. Control cells were inoculated two days before METTL3-high cells. When the tumor sizes were similar, volume-matched mice received everolimus (50 µg/day intragastrically) or solvent for 17 days and were sacrificed at day 18. Mice were randomly allocated to each group with no blinding.

### In silico analyses

All datasets used in this study were derived from public databases. Enrichment analyses were performed by DAVID (https://david.ncifcrf.gov) and TAM (http://www.cuilab.cn/tam). Prediction of miRNA targets was performed by the online tool miRDB (http://mirdb.org/).

### Statistical analyses

Data were from at least three independent experiments unless otherwise specified. Variations within each group were estimated and were all statistically compared. Differences between groups were calculated by Student’s *t*-tests. Univariate analyses were performed by Chi-squared tests. Survival data were compared by log-rank tests. Correlations were analyzed by linear regression. All the statistical tests were two-tailed. *P* values < 0.05 were considered statistically significant. Data were presented as mean or mean ± standard deviation (SD).

## Results

### Elevated m^6^A is mainly regulated by its “writer” METTL3 in gastric cancer

**Fig. 1:**
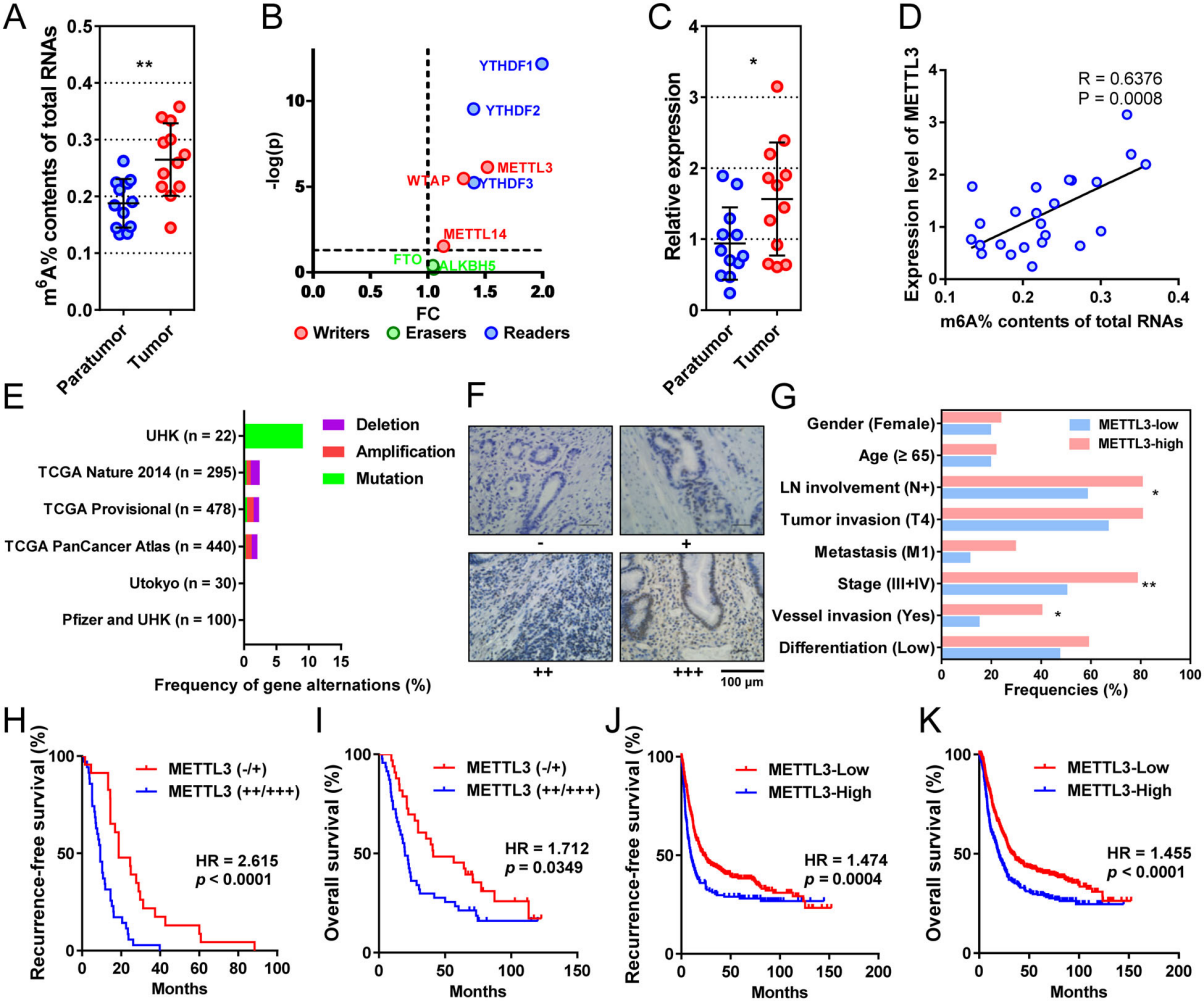
METTL3 is overexpressed in gastric cancer and associated with poor prognosis in GC. **a** m^6^A levels of total RNA derived from 12 pairs of cancerous and noncancerous tissues. **b** Differential expression (gastric cancer *vs*. normal tissue) of the m^6^A regulators in the TCGA-STAD database. The colors indicate the functional subgroups of the proteins. **c** Expression of METTL3 mRNA in 12 pairs of cancerous and noncancerous tissues. **d** Correlation between METTL3 mRNA levels (y-axis) and percentage of m^6^A content in total RNA (*x*-axis) in our clinical cohorts (*n* = 24). **e** Genetic changes of METTL3 in gastric cohorts from online databases. Original data were extracted from cBioPortal. **f** Representative IHC staining of METTL3 in GC tissues (100×). One representative sample of staining intensity (-), (+), (++), and (+++) were shown. Scale bars represent 50 μm. **g** Clinicopathologic features of patients with METTL3-low (*n* = 36) and high tumors (*n* = 51). **h**, **i** Kaplan–Meier plots of RFS (*n* = 58) and OS (*n* = 80) of patients with METTL3-low and high tumors according to IHC staining intensity. Data were derived from a gastric cancer cohort followed-up by us. **j**, **k** Kaplan–Meier plots of RFS (*n* = 641) and OS (*n* = 876) of patients with METTL3-low and high tumors according to mRNA levels. Data were interrogated from the GEO datasets GSE14210, GSE15459, GSE22377, GSE29272, GSE38749, GSE51105, and GSE62254 with the probe 209265_s_at. Data are presented as mean ± SD. *P < 0.05; **P < 0.01.

To explore the features of m^6^A in gastric cancer, we first compared the levels of m^6^A on total RNAs from 12 pairs of cancerous and adjacent tissues. The m^6^A levels were significantly elevated in tumor tissues compared with their adjacent tissues (Fig. 1a). To determine the key regulators, we performed differential expression analysis on the m^6^A “writers”, “erasers”, and “readers” from gastric cancer and normal tissues in The Cancer Genome Atlas Stomach Adenocarcinoma (TCGA-STAD) database. The “writers” and “readers” were all overexpressed in gastric cancer, whereas “erasers” were expressed similarly in tumors and controls (Fig. 1b). Based on this, we then focused on the most differentially expressed “writer”, METTL3, for further study.

METTL3 RNA level was significantly higher in cancerous tissues than that in adjacent tissues (Fig. 1c). In addition, METTL3 RNA positively correlated with total RNA m^6^A levels in our cohort (Fig. 1d). When mutations were considered in the previously published cohorts, genetic changes in METTL3 only accounted for 2.12% of total patients (Fig. 1e).

### METTL3 overexpression indicates adverse pathological features and poor outcome in gastric cancer

To determine the clinical relevance of METTL3, we tested METTL3 expression in gastric cancer samples of 87 patients who had received radical or palliative gastrectomy (Fig. 1f). METTL3 was mainly distributed in the nucleus of tumor cells. According to the staining intensity, 12 negative (−), 24 weak positive (+), 32 medium positive (++), and 19 strong positive cases (+++) were observed. METTL3 expression significantly correlated with AJCC staging (the eighth edition, *P* = 0.0056), lymph node metastasis (*P* = 0.0251), and vascular invasion (*P* = 0.0346), but did not correlate with gender, age, differentiation, and tumor invasion (Table 1, Fig. 1g).

**Table 1:** Correlation of clinicopathologic features with METTL3 expression in gastric cancer tissues.

	Number^a^	METTL3 expression		*P* value^b^
Clinicopathological features		Low^a^	High^a^	
Gender				0.6497
Male	68 (78.16%)	29 (42.65%)	39 (57.35%)	
Female	19 (21.84%)	7 (36.85%)	12 (63.15%)	
Age				0.8096
≥65	18 (20.69%)	7 (38.89%)	11 (61.11%)	
<65	69 (79.31%)	29 (42.03%)	40 (57.97%)	
Stage				0.0056**
I + II	29 (33.33%)	18 (62.07%)	11 (39.93%)	
III + IV	58 (66.67%)	18 (31.03%)	40 (68.97%)	
Tumor invasion				0.5317
T1 or T2 or T3	30 (34.48%)	12 (40.00%)	18 (60.00%)	
T4	57 (65.52%)	24 (42.11%)	33 (57.89%)	
Lymph node involvement				0.0251*
Yes	62 (71.26%)	21 (33.87%)	41 (66.13%)	
No	25 (28.74%)	15 (60.00%)	10 (40.00%)	
Metastasis				0.0639
M0	68 (78.16%)	32 (47.06%)	36 (52.94%)	
M1	19 (21.84%)	4 (21.05%)	15 (78.95%)	
Differentiation				0.2849
Poorly	47 (54.02%)	17 (36.17%)	30 (63.83%)	
Well	40 (45.98%)	19 (47.50%)	21 (52.50%)	
Microvascular invasion				0.0346*
Present	16 (28.07%)	4 (25.00%)	12 (75.00%)	
Absent	41 (71.93%)	23 (56.10%)	18 (43.90%)	

^a^Data are presented as numbers (proportions).

^b^*P* values were calculated by Chi-square test or Fisher’s exact test.

*P < 0.05; **P < 0.01.

Patients with METTL3 overexpression (++/+++) had higher risks of recurrence (hazard ratio (HR) = 2.615, 95% confidence interval (CI) = 1.934–5.623, Fig. 1h) and death (HR = 1.712, 95% CI = 1.042–2.799, Fig. 1i), with significantly shorter median recurrence-free survival (mRFS, 9.3 vs. 18.7 months, *P* = 0.0002, Fig. 1h) and mOS (19.6 vs. 41.1 months, *P* = 0.0349, Fig. 1i) than those with low METTL3 expression (−/+). In addition, the prognostic value of METTL3 was also confirmed by the online tool Kaplan–Meier plotter. In this database, the METTL3-high patients had higher risks of recurrence (HR = 1.474, 95% CI = 1.211–1.955, Fig. 1j) and death (HR = 1.455, 95% CI = 1.238–1.841, Fig. 1k), with shorter mRFS (9.7 vs. 19.8 months, *P* = 0.0004, Fig. 1j) and mOS (19.8 vs. 33.2 months, *P* < 0.0001, Fig. 1k) than the METTL3-low gastric cancer patients.

### METTL3 promotes gastric cancer cell proliferation and tumor growth

**Fig. 2:**
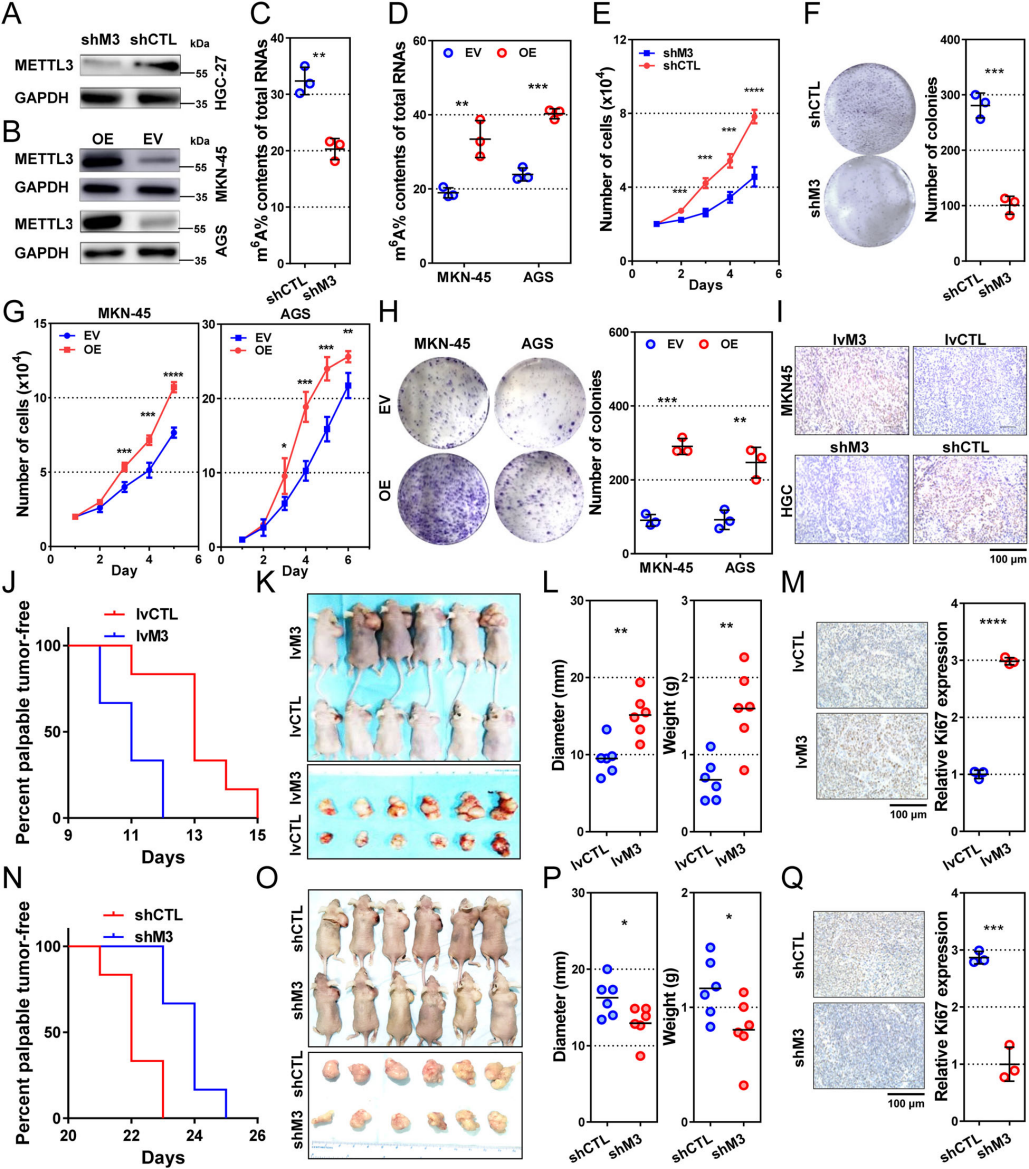
METTL3 promotes gastric cancer cell proliferation and tumor growth. **a** METTL3 expression in HGC-27 cells transfected with lentivirus encoding shRNA against METTL3 (shM3) and control (shCTL). **b** METTL3 levels in MKN-45 and AGS cells transfected with overexpressing plasmid (OE) or empty vector (EV). **c**, **d** Percentage of m^6^A content in total RNA in METTL3-reducing HGC-27 and METTL3-overexpressing MKN-45 and AGS cells. **e** Proliferation curves of METTL3-reducing HGC-27 cells. **f** Colony-formation assays of METTL3-reducing HGC-27 cells. Representative plates are shown on the left, and quantification on the right. **g** Proliferation of METTL3-overexpressing MKN-45 and AGS cells. **h** Colony-formation assays of METTL3-overexpressing MKN-45 and AGS cells. *n* = 3 for each group in **c**–**h**. **i** METTL3 expression in xenograft tumors derived from stable cell lines transfected with lentivirus encoding METTL3 (lvM3, MKN-45 cells), shM3 (HGC-27 cells), and their corresponding controls (lvCTL and shCTL). **j** Kaplan–Meier plots of palpable tumor-free survival in METTL3-high (*n* = 6) and control (*n* = 6) MKN-45 cell xerographs. **k** Tumor-harboring mice (top) and xenograft tumors (bottom) 3 weeks post-implantation of METTL3-overexpressing and control MKN-45 cells. **l** Diameter (left) and weight (right) of METTL3-high and control MKN-45 tumors. Each dot represents one sample. **m** Ki67 levels of METTL3-high and control MKN-45 tumors. **n** Kaplan–Meier plots of palpable tumor-free survival in METTL3-low (*n* = 6) and control (*n* = 6) HGC-27 xerographs. **o** Tumor-harboring mice (top) and xenograft tumors (bottom) 35 days post-inoculation of METTL3-reducing and control HGC-27 cells. **p** Diameter (left) and weight (right) of METTL3-low and control HGC-27 tumors. Each dot represents one sample. **q** Ki67 levels of METTL3-low and control HGC-27 tumors. Data are presented as mean ± SD. *P < 0.05; **P < 0.01; ***P < 0.001; ****P < 0.0001.

To investigate the biological role of METTL3 in gastric cancer, we established METTL3-downregulated (Fig. 2a and supplementary Fig. S1a) and -upregulated cells (Fig. 2b) using lentivirus containing shRNA and plasmids containing METTL3, respectively. Compared to those in control cells, the m^6^A levels of total RNA were significantly decreased in METTL3-downregulated cells (*P* = 0.0024 and 0.0015, Fig. 2c and supplementary Fig. S1b) and elevated in METTL3-upregulated cells (*P* = 0.0082 and 0.0002, Fig. 2d).

In cell models, METTL3 downregulation impeded cell proliferation (all *P* < 0.05 from day 2, Fig. 2e and supplementary Fig. S1c) and colony formation (*P* = 0.0004 and 0.0010, Fig. 2f and supplementary Fig. S1d). Accordingly, METTL3 overexpression stimulated cell proliferation (all *P* < 0.05 from day 3, Fig. 2g) and colony-forming efficiencies (*P* = 0.0002 and 0.0055, Fig. 2h).

Subsequently, subcutaneous xenograft models were established for in vivo studies. The median times to form palpable tumors were 11.0 and 13.5 days in METTL3-overexpressed and control cells, respectively (*P* = 0.0055, Fig. 2i, j). Three weeks after subcutaneous injection, tumors from METTL3-overexpressing cells were remarkably larger (*P* = 0.0028, Fig. 2k, l) and heavier (*P* = 0.0027, Fig. 2l) than tumors from control cells. In addition, Ki67 was also overexpressed in METTL3-high tumors (*P* < 0.0001, Fig. 2m). In mice with METTL3-low tumors, the median time to form touchable tumors was significantly longer than that in control mice (24.0 vs. 22.0 days, *P* = 0.0055, Fig. 2i, n). Further, METTL3-low tumors were significantly smaller (*P* = 0.0369, Fig. 2o, p) and had lower weights (*P* = 0.0421, Fig. 2p) and Ki67 expression (*P* = 0.0005, Fig. 2q) than the control group.

### METTL3 promotes gastric cancer cell migration, invasion, and peritoneal metastasis

**Fig. 3:**
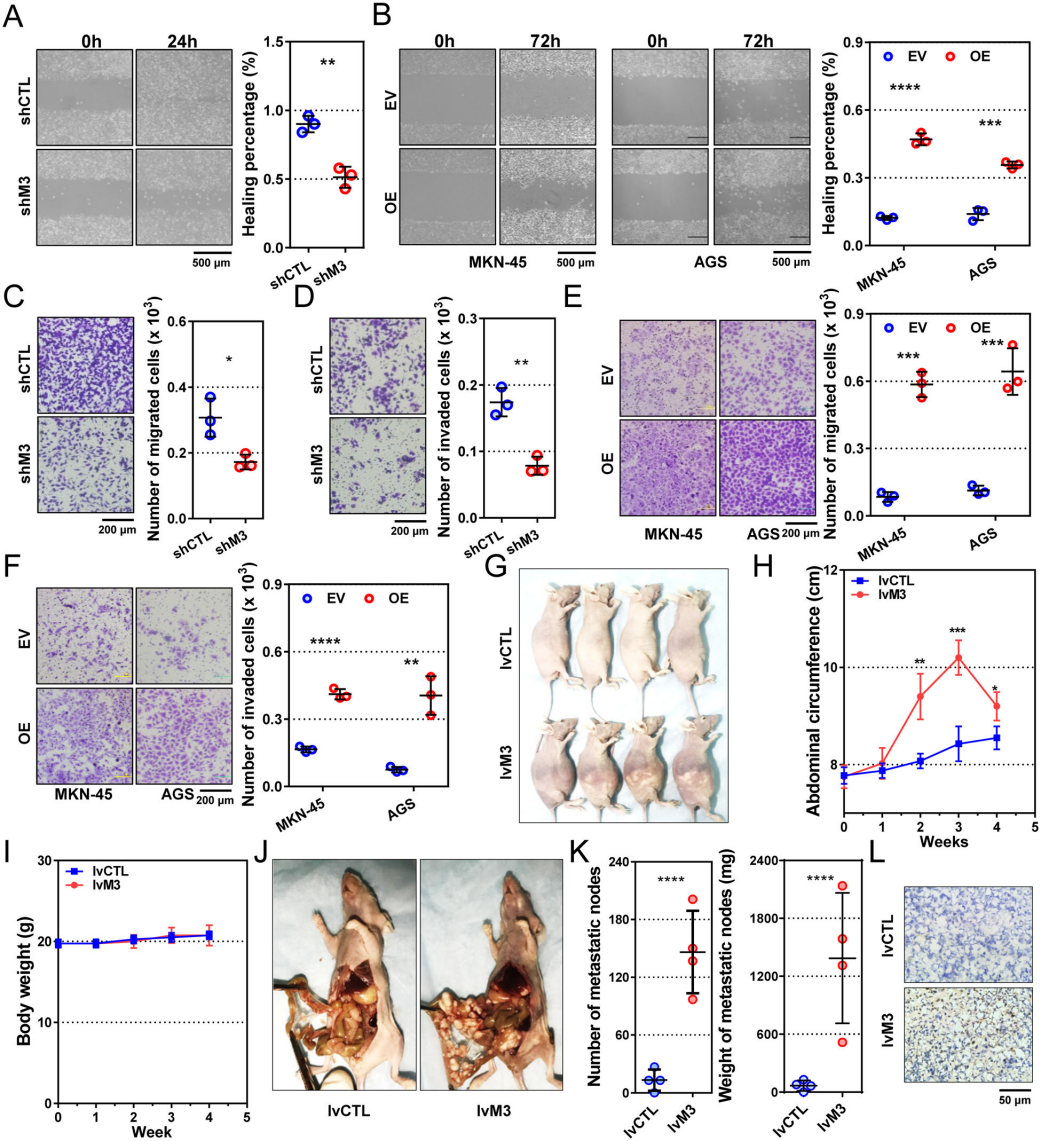
METTL3 promotes gastric cancer cell motility and peritoneal colonization. **a**, **b** Wound-healing assays of METTL3-reducing (shM3) HGC-27, METTL3-overexpressing (OE) MKN-45, and AGS, and their corresponding control (shCTL and EV) cells. **c**, **d** Migration and invasion assays of METTL3-reducing HGC-27 cells. **e**, **f** Migration and invasion assays of METTL3-overexpressing MKN-45 and AGS cells. Representative images on the left, and quantification bars on the right in **a**–**f** (*n* = 3 for each group). **g** Mice with peritoneal implant nodes derived from METTL3-overexpressing (lvM3; *n* = 4) and control (lvCTL; *n* = 4) MKN-45 cells. **h**, **i** Curves of abdominal circumferences and body weights of the peritoneal metastasis models. **j** Representative pictures of the peritoneal implants from stable METTL3-overexpressing and control MKN-45 cells. **k** Number (left) and weight (right) of implanted nodes in peritonea of mouse models. Each dot represents one sample. **l** Representative pictures of METTL3 expression in peritoneal nodes by IHC. Data are presented as mean ± SD. *P < 0.05; **P < 0.01; ***P < 0.001; ****P < 0.0001.

In wound-healing assays, METTL3 downregulation reduced migration distances remarkably (*P* = 0.0023 and 0.0005, Fig. 3a and supplementary Fig. S1e), while METTL3 overexpression increased the migration distances (*P* < 0.0001 and =0.0003, Fig. 3b). In Transwell assays, the METTL3-low cells showed decreased migration (*P* = 0.0199 and 0.0061, Fig. 3c and supplementary Fig. S1f) and invasion (*P* = 0.0029 and 0.0003, Fig. 3d and supplementary Fig. S1g) compared to the control cells. Accordingly, METTL3 overexpression significantly augmented cell migration (*P* = 0.0001 and 0.0010, Fig. 3e) and invasion (*P* < 0.0001 and =0.0028, Fig. 3f) in two cell models.

Next, we established a gastric cancer peritoneal metastasis model by intraperitoneal injection of tumor cells (Fig. 3g). The abdominal circumferences of the mice bearing METTL3-high cells increased rapidly and became larger than those of the mice bearing control cells from the second week (all *P* < 0.05, Fig. 3h). However, body weights were not significantly different between the two groups (Fig. 3i). Most mice with METTL3-high cells showed palpable masses on the abdominal wall, which was not observed in the control group (Fig. 3g). When dissected four weeks later, the mice bearing METTL3-high cells possessed more and larger peritoneal-implanted nodules (both *P* < 0.0001, Fig. 3j, k). They were mainly distributed in the mesentery and omentum (Fig. 3j), while few grew on the surface of the liver or spleen. METTL3 overexpression in implanted peritoneal nodes was confirmed by IHC (Fig. 3l).

### METTL3 promotes tumor progression by facilitating biogenesis of miR-17-92 cluster

**Fig. 4:**
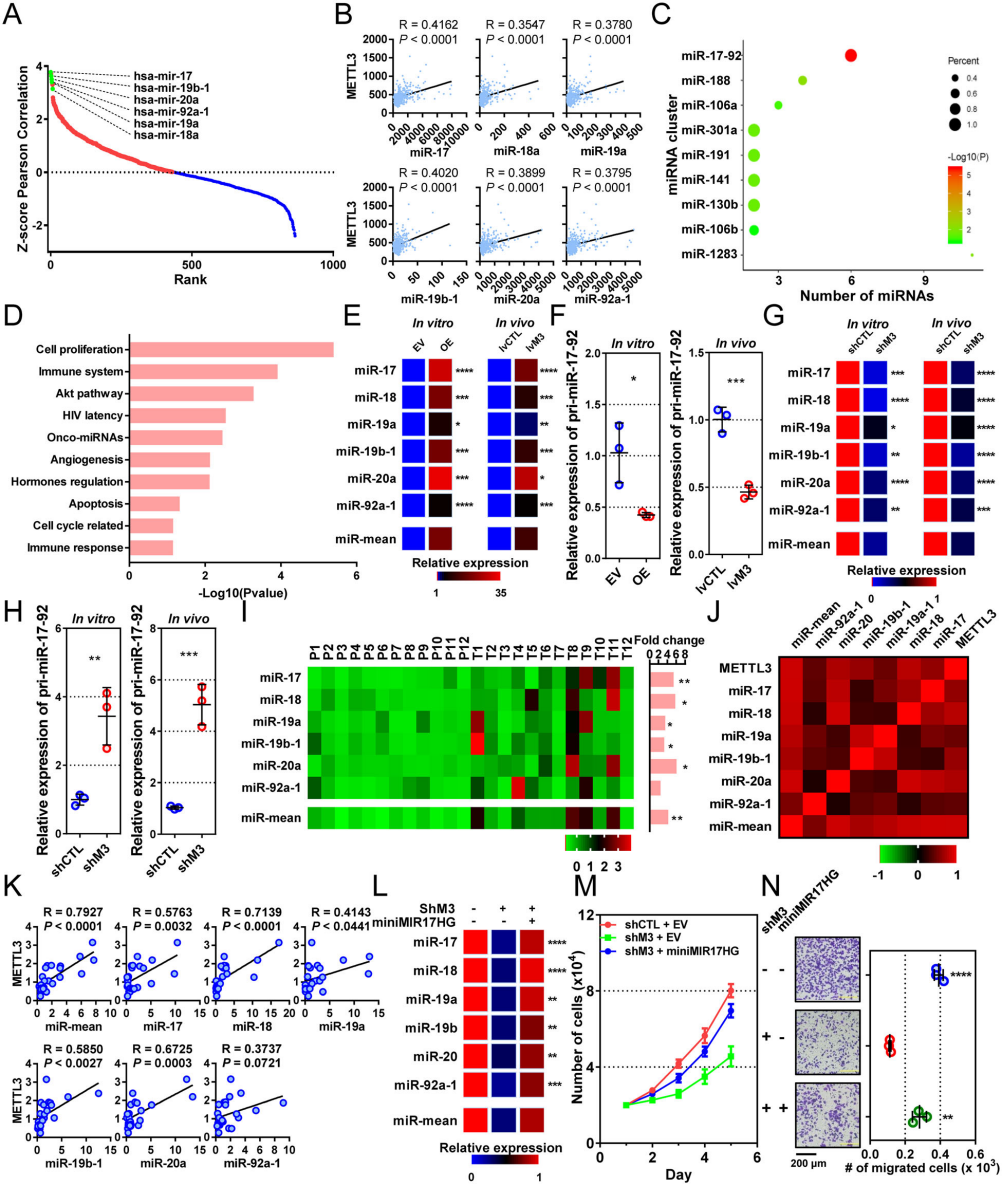
METTL3 promotes tumor progression by facilitating biogenesis of miR-17-92 cluster. **a** Correlation between METTL3 mRNA and all miRNAs in the TCGA-STAD database. *Pearson* correlation coefficients were transformed into Z-scores (*y*-axis) and ranked in descending order (*x*-axis). Blue and red dots represent negatively and positively METTL3-correlated miRNAs, respectively. Green dots represent the members of the miR-17-92 cluster. **b** Correlations between METTL3 (*y*-axis) and members of the miR-17-92 cluster (*x*-axis). Data were derived from the TCGA-STAD database. **c** MiRNA cluster enrichment results of the top METTL3-correlated miRNA (*n* = 76). The *y*-axis indicates the miRNA clusters that miRNAs were enriched in, and the *x*-axis indicates the numbers of METTL3-correlated miRNAs enriched in this cluster. Dot sizes indicate percentage of the METTL3-correlated miRNA in total miRNAs of the cluster, and colors indicate the *P* values of the enrichment. **d** Top 10 functional terms from METTL3-correlated miRNA enrichment. **e**, **f** Relative expressions of the miR-17-92 cluster and pri-miR-17-92 in METTL3-overexpressing (OE and lvM3) and control (EV and lvCTL) MKN-45 cells in vitro and in vivo. MiR-mean indicates mean value of the miR-17-92 cluster. **g**, **h** Relative expression of the miR-17-92 cluster and pri-miR-17-92 in METTL3-reducing (shM3) and control (shCTL) HGC-27 cells in vitro and in vivo. *n* = 3 for each group in **e**–**h**. **i** Relative expression of miR-17-92 cluster in fresh cancerous (T1–T12) and noncancerous tissues (P1–P12). Relative expression was indicated by the heatmap (left). Fold change (cancerous vs. noncancerous tissues) and statistical significance were shown on the right. **j** Correlation among METTL3 mRNA and miRNA miR-17-92 clusters. Correlation coefficients indicated by the heatmap. **k** Correlation between METTL3 mRNA (*y*-axis) and miR-17-92 cluster (*x*-axis) shown with scatter plots. **l** Expression of miR-17-92 cluster in HGC-27 cells expressing shM3 rescued by miniMIR17HG and empty vector (EV) transfection. **m**, **n** Proliferation and migration assays of HGC-27 cells expressing shM3 rescued by miniMIR17HG expression. The indicated significances were for comparisons between METTL3-reducing cells and those transfected with miniMIR17HG. Data are presented as mean ± SD. *P < 0.05; **P < 0.01; ***P < 0.001; ****P < 0.0001.

To decipher the mechanisms of METTL3 in tumor growth and metastasis, we performed correlation analysis between METTL3 mRNA and all the other mRNA, miRNA, and long ncRNA in the TCGA-STAD database. Among the top 7 METTL3-correlated miRNAs, 6 (miR-17, 19b-1, 20a, 92a-1, 19a, and 18a) were derived from one miRNA cluster, the miR-17-92 cluster, which shared a primary miRNA, the pri-miR-17-92 (the transcript of *MIR17HG*, Fig. 4a, b). Meanwhile, none of these miRNAs targeted METTL3 mRNA, as predicted by miRDB. By cluster enrichment analysis of miRNA with software TAM, 76 METTL3-correlated miRNAs (correlation coefficients >0.2 and FDR < 4 × 10^−6^) were also significantly enriched in the miR-17-92 cluster (*P* < 0.0001, Fig. 4c). In terms of function, these METTL3-correlated miRNAs were enriched in several cancer-associated pathways, such as cell proliferation, immunity, AKT pathway, onco-miRNA, angiogenesis, apoptosis, and cell cycle (Fig. 4d).

We then examined the expression levels of the miR-17-92 cluster and pri-miR-17-92 in cells and xenograft tumors. Both in vitro or in vivo, METTL3 overexpression significantly increased levels of all six miRNAs (all *P* < 0.05, Fig. 4e) and reduced the level of pri-miR-17-92 (*P* = 0.0229 in vitro and *P* = 0.0009 in vivo, Fig. 4f), while METTL3 downregulation reduced all miRNAs (all *P* < 0.05, Fig. 4g) and increased the level of pri-miR-17-92 (*P* = 0.0078 in vitro and *P* = 0.0009 in vivo, Fig. 4h).

The correlation between METTL3 and the miRNA-17-92 cluster was also confirmed in our clinical samples. Levels of miR-17, 18, 19a, 19b-1, and 20a, as well as the average level of all six miRNAs (miR-mean), were significantly elevated in tumors compared with those in adjacent tissues (*P* < 0.05 for all, Fig. 4i). The expression of each member of the miRNA-17-92 cluster was positively correlated with the others (Fig. 4j). In addition, most members (except miR-92a-1) and miR-mean correlated positively with the level of METTL3 mRNA with statistical significance (*P* < 0.05 for all, Fig. 4j, k).

To verify whether METTL3 exerts the onco-promoting role by facilitating pri-miR-17-92 maturation, we constructed a miniMIR17HG plasmid to force expression of 6 miRNAs for rescue studies^14^. This construct contains all miRNAs from the miR-17-92 cluster but no flanking sequences and can be rapidly processed into mature miRNAs. Transfection of miniMIR17HG in METTL3-knocked-down cells significantly raised all miRNAs (all *P* < 0.01) to similar levels of control cells (Fig. 4l). In addition, miniMIR17HG partially rescued cell proliferation (*P* < 0.05 from day 2, Fig. 4m) and cell migrations (*P* = 0.0019, Fig. 4n).

### METTL3 facilitates pri-miR-17-92 processing by m^6^A modification on its A879 locus

**Fig. 5:**
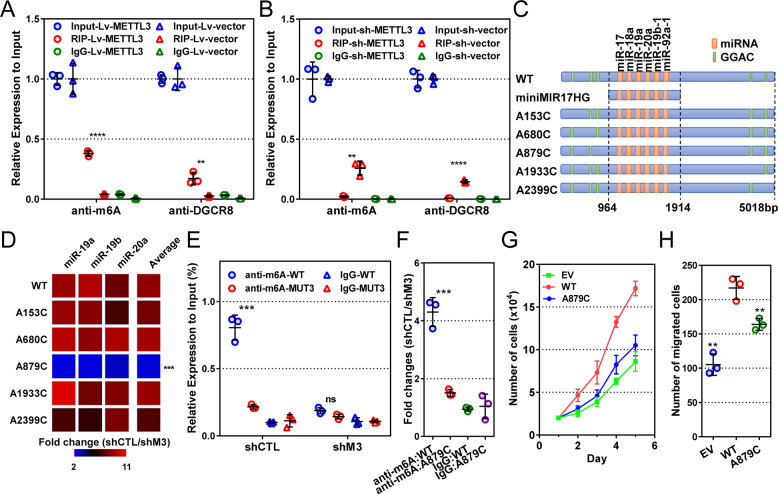
METTL3 facilitates pri-miR-17-92 processing by m^6^A modification on the A879 locus. **a** Results of m^6^A-RIP and DGCR8-RIP in METTL3-overexpressing (lvM3) and control (lvCTL) MKN-45 cells. **b** Results of m^6^A-RIP and DGCR8-RIP in METTL3-reducing (shM3) and control (shCTL) HGC-27 cells. Input RNA and RNA pulled down by specific (anti-m^6^A or anti-DGCR8) antibodies (Sab) or polyclonal immunoglobulin G (IgG) were quantified by RT-PCR, and their ratios to corresponding inputs were shown in **a**, **b**. **c** Schematic diagram of the wild type pri-miR-17-92 (WT), miniMIR17HG, and pri-miR-17-92 with point mutations. “GGAC” motifs were not displayed in the diagram when mutated into “GGCC”. **d** Fold change (control vs. METTL3-reducing HGC-27 cells) of miR-19a, 19b, 20a, and their mean values when cells were transfected with vectors encoding pri-miR-17-92-WT or those with point mutations. Statistical significance is for comparisons between cells transfected with pri-miR-17-92-WT and those with mutations. **e** m^6^A-RIP in METTL3-reducing and control HGC-27 cells transfected with pri-miR-17-92-WT, A879, or empty vector (EV). RNAs pulled down by anti-m^6^A or IgG were quantified by RT-PCR, and their ratios to inputs are displayed. Statistical significance is for comparisons between the m^6^A-RIP data from cells transfected with WT and A879C. **f**, **g** Proliferation and migration assays of HGC-27 cells overexpressing pri-miR-17-92-WT, A879C, and EV. The indicated significances were for comparisons between cells overexpressing pri-miR-17-92-WT and A879C. *n* = 3 for each group. Data are presented as mean ± SD. ^ns^*P* > 0.05; **P < 0.01; ***P < 0.001; ****P < 0.0001.

To explore the mechanisms by which METTL3 regulates pri-miR-17-92 processing, we examined its function on m^6^A regulation of pri-miR-17-92 and pri-miR-17-92-DGCR8 binding by RNA RIP. METTL3 overexpression significantly increased the m^6^A modification on pri-miR-17-92 (*P* < 0.0001, Fig. 5a). Besides, METTL3 remarkably facilitated DGCR8 binding to pri-miR-17-92 (*P* = 0.0077, Fig. 5a). Consistently, METTL3 downregulation repressed m^6^A modification on pri-miR-17-92 (*P* = 0.0021) and DGCR8-pri-miR-17-92 binding (*P* < 0.0001, Fig. 5b).

To identify the specific sites of m^6^A on pri-miR-17-92 by METTL3, we analyzed its sequence and found five “GGAC” motifs that were potentially recognized by METTL3 (Fig. 5c). Five plasmids with adenine to cytosine mutation in these motifs were constructed and transfected into cells (Fig. 5c). Compared to the wild type plasmid, A879C mutation hindered miRNA biogenesis significantly, while the others did not (Fig. 5d). Also, A879C mutation remarkably reduced the precipitated pri-miR-17-92 by anti-m^6^A antibody in control cells with normal expression of METTL3 (*P* = 0.0004), but this difference was not observed in METTL3-knocked-down cells (Fig. 5e). In another aspect, the wild type pri-miR-17-92 with m^6^A in control cells was 4.3 times as much as that in METTL3-low cells (Fig. 5f). In contrast, the difference of pri-miR-17-92-A879C with m^6^A was only 1.5 times in these two cell lines, indicating a key role of A879 in the METTL3-mediated pri-miR-17-92 processing (Fig. 5f).

In cell proliferation assays, cells transfected with wild type pri-miR-17-92 showed superior proliferation ability to both pri-miR-17-92-A879C-expressing and control cells from day 1 to 4 (all *P* < 0.05 from day 2, Fig. 5g). In addition, cells transfected with pri-miR-17-92-A879C also showed inferior migrating ability compared to those transfected with wild type pri-miR-17-92 (*P* = 0.0082, Fig. 5h).

### METTL3 inhibits PTEN/TMEM127 expression and activates AKT/mTOR pathway by facilitating biogenesis of miR-17-92 cluster

**Fig. 6:**
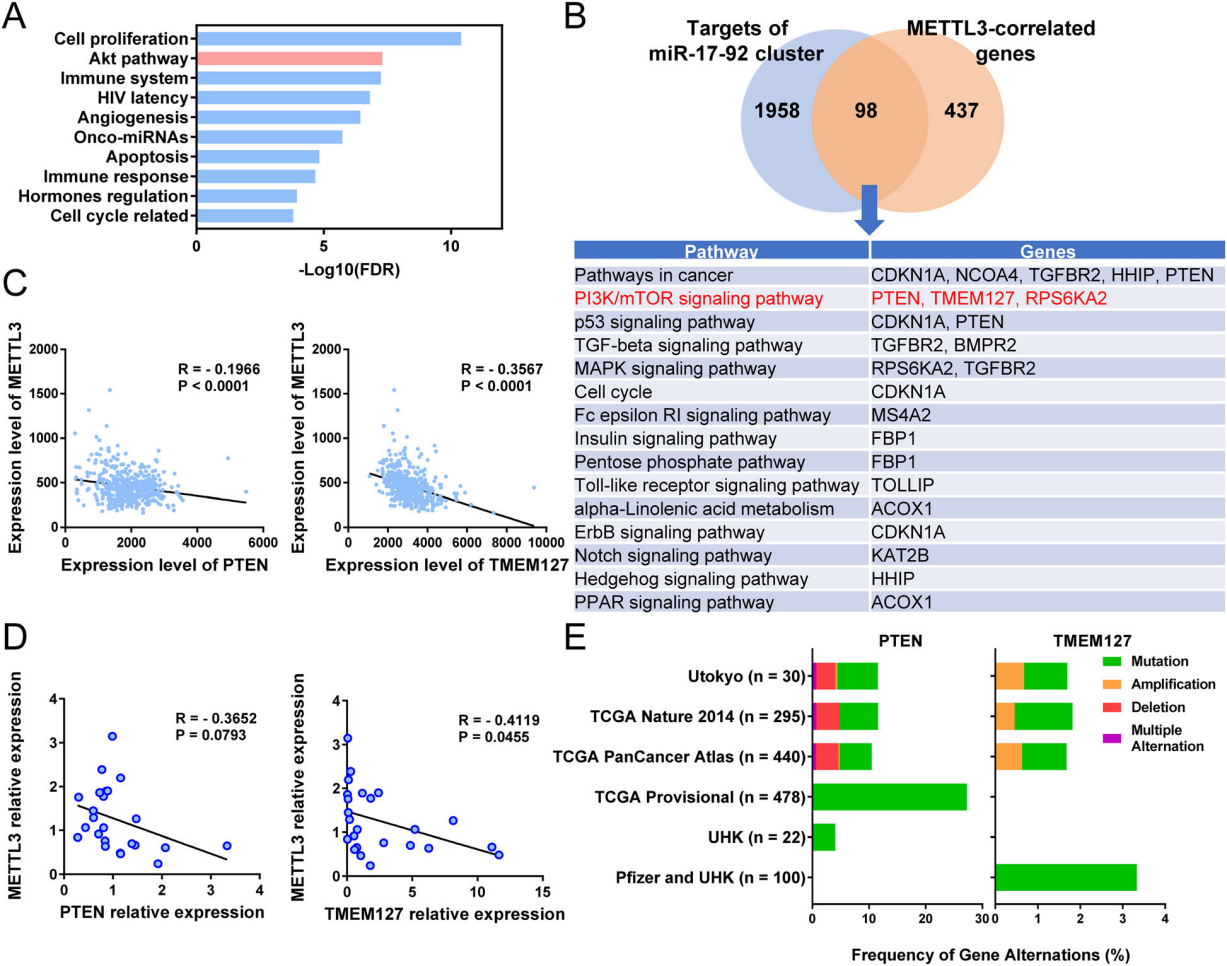
PTEN and TMEM127 are potential targets of METTL3/miR-17-92 cluster. **a** Top 10 terms from miRNA functional enrichment of the miR-17-92 cluster. **b** KEGG analysis of the METTL3- and miR-17-92 cluster-regulated genes. (Upper) Number of potential target genes of the miR-17-92 cluster, METTL3-correlated genes, and their intersections. (Lower) Table with the KEGG results (pathways and involved genes) of the intersectional genes. **c** Correlation between METTL3 (*y*-axis) and PTEN or TMEM127 (*x*-axis) in the TCGA-STAD database. **d** Correlation between METTL3 (*y*-axis) and PTEN or TMEM127 (*x*-axis) in our clinical cohorts. **e** Genetic change of *PTEN* and *TMEM127* in gastric cohorts from online databases. Original data were extracted from cBioPortal. FDR, false discovery rate adjusted *P* value.

To determine the downstream targets of the miR-17-92 cluster in gastric cancer progression, we performed miRNA cluster enrichment analysis and found the members of the miR-17-92 cluster were significantly enriched in several cancer-related terms, including cell proliferation, AKT pathway, angiogenesis, onco-miRNAs, and apoptosis (Fig. 6a). Also, we obtained 2056 target genes of the miR-17-92 cluster predicted by miRDB and 525 mRNAs that negatively correlated with METTL3 with a correlation coefficient < −0.18 in the TCGA-STAD database. The two gene pools shared a total of 98 genes, which were enriched in PI3K/mTOR, p53, TGF-β, and MAPK pathways by KEGG analysis (Fig. 6b). Thus, we chose the members that were clustered in the PI3K/mTOR pathway, PTEN and TMEM127, for further study.

In the TCGA-STAD databases, both PTEN and TMEM127 were negatively correlated with METTL3 expression with statistical significance (both *P* < 0.0001, Fig. 6c). These negative correlations between METTL3 mRNA and PTEN/TMEM127 mRNA were confirmed in our clinical samples by quantitative RT-PCR (Fig. 6d). Nonsynonymous mutation and copy number variations (CNVs) of *PTEN* occurred in about 10.6% of gastric patients (Fig. 6e). Among them, all CNVs of *PTEN* were deletion instead of amplification (Fig. 6e). In contrast, nonsynonymous mutations and CNVs of *TMEM127* only occurred in 1.6% of the patients, and all CNVs were amplification (Fig. 6e).

**Fig. 7:**
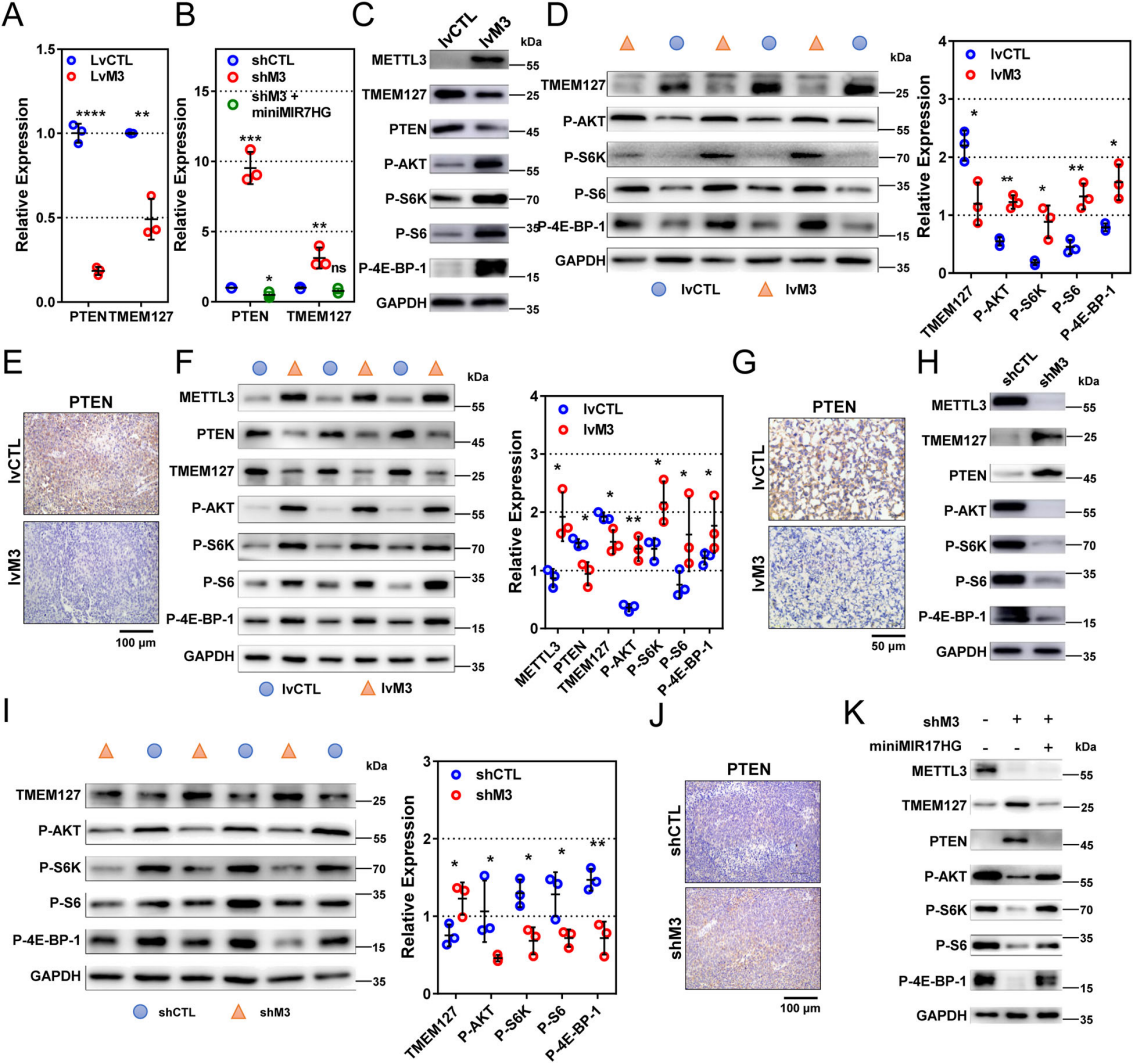
METTL3 modulates PTEN/TMEM127 and activates AKT/mTOR pathway by facilitating processing of pri-miR-17-92. **a**, **b** Relative mRNA of PTEN and TMEM127 in METTL3-overexpressing MKN-45 (lvM3), METTL3-reducing HGC-27 (shM3), and control cells (lvCTL and shCTL) by RT-PCR. **c** Protein levels in METTL3-overexpressing and control MKN-45 cells by western blot. **d**, **e** Protein levels in METTL3-high (*n* = 3) and control (*n* = 3) xenograft MKN-45 tumors by western blot or IHC. Quantification is derived from data of three mice. **f**, **g** Protein levels in METTL3-high (*n* = 3) and control (*n* = 3) peritoneal implants by western blot and IHC. **h** Protein levels in METTL3-reducing and control HGC-27 cells by western blot. **i**, **j** Protein levels in METTL3-low (*n* = 3) and control (*n* = 3) xenograft HGC-27 tumors by western blot and IHC. **k** Protein levels in control, METTL3-reducing, and METTL3-reducing/miR-17-92 cluster-expressing HGC-27 cells by western blot. Data are presented as mean ± SD. *P < 0.05; **P < 0.01; ***P < 0.001; ****P < 0.0001.

By RT-PCR, METTL3 overexpression significantly reduced mRNA levels for PTEN (*P* < 0.0001) and TMEM127 (*P* = 0.0019, Fig. 7a), while METTL3 downregulation elevated their mRNA levels (*P* = 0.0002 and 0.0083, Fig. 7b). Furthermore, forced expression of miniMIR17HG in METTL3-reducing cells reversed (of PTEN, *P* = 0.0124) or diminished (of TMEM127, *P* = 0.0929) the differences (Fig. 7b). By western blot and IHC, both PTEN and TMEM127 were remarkably reduced in METTL3-high cells, subcutaneous xenografts, and peritoneal implants (Fig. 7c, g). Accordingly, when METTL3 was reduced, PTEN and TMEM127 were significantly elevated in cells and subcutaneous xenografts (Fig. 7h–j). What is more, the overexpression of PTEN and TMEM127 caused by METTL3-knockdown was reversed by miniMIR17HG (Fig. 7k).

We continued to investigate whether METTL3/miR-17-92 cluster influenced the AKT/mTOR pathway activation. In METTL3-high cells, subcutaneous xenografts, and peritoneal metastatic nodes, the phosphorylation levels of AKT/mTOR pathway-related proteins, including AKT, S6K, S6, and 4E-BP1, were all upregulated (Fig. 7c, d, f), which indicated the activation of the AKT/mTOR pathway. In the METTL3-reducing cells and xenograft tumors, phosphorylation levels of AKT, S6K, S6, and 4E-BP1 were significantly decreased (Fig. 7h, i). Further, forced expression of miniMIR17HG counteracted the AKT/mTOR pathway inactivation by METTL3-knocked-down (Fig. 7k).

### Gastric cancer with METTL3 overexpression is more sensitive to everolimus

**Fig. 8:**
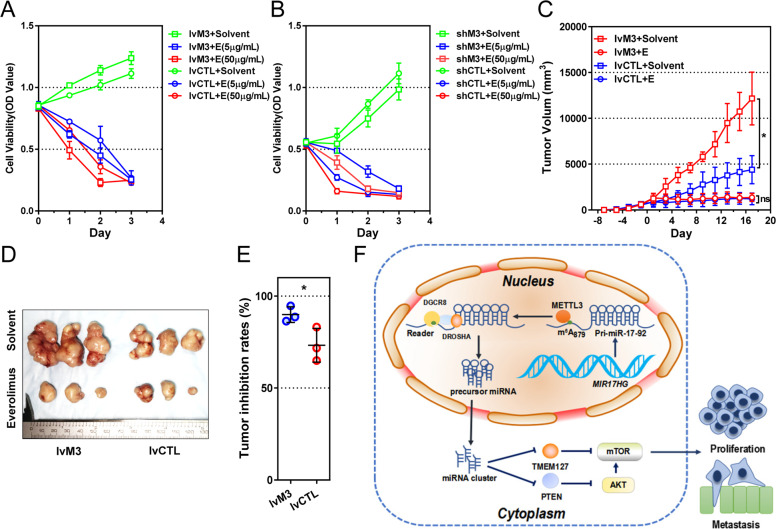
Gastric cancer with METTL3 overexpression is more sensitive to everolimus. **a**, **b** Cell viability assays of METTL3-overexpressing MKN-45 (lvM3), METTL3-reducing HGC-27 (shM3), and control (lvCTL and shCTL) cells, with (5 or 50 µg/mL) or without everolimus (E). **c** Tumor volume of mice carrying METTL3-high or control tumors and fed with everolimus or solvent. Mice were pre-inoculated with MKN-45 tumor cells, administrated with everolimus or solvent daily from day 0 to 16, then sacrificed at day 17. **d** Photos of xenograft tumors when mice were sacrificed. **e** Tumor inhibition rate of everolimus in METTL3-high and control tumors. Tumor inhibition rate = 1 − the tumor weight with everolimus/the corresponding tumor weight with solvent. **f** Overview of METTL3/m^6^A-mediated miRNA cluster biogenesis and AKT/mTOR pathway activation in gastric cancer development. *n* = 3 for each group. Data are presented as mean ± SD. *P < 0.05.

Due to the remarkable impact of the METTL3/miR-17-92 cluster on the AKT/mTOR pathway, we explored whether everolimus, an mTOR inhibitor, could inhibit the onco-promoting role of METTL3. Cell viability was measured in cell models with different levels of METTL3 after treatment with solvent or different concentrations of everolimus. Without everolimus, METTL3 overexpression increased cell viability (*P* < 0.05 from day 1, Fig. 8a), whereas METTL3 downregulation decreased cell viability (*P* < 0.05 from day 1, Fig. 8b), compared to their corresponding controls. Addition of everolimus remarkably suppressed cell viability, regardless of its concentrations or METTL3 expression (Fig. 8a, b). However, METTL3-overexpressing cells showed more sensitivity to everolimus in a dose-dependent manner than the control cells (*P* < 0.05 at day 2 and 3, Fig. 8a). In accordance, the METTL3-low cells showed lower sensitivity to everolimus in both concentrations than the control cells (*P* < 0.05 at day 2 and 3, Fig. 8b).

With in vivo models, everolimus was effective in reducing tumor volumes in both METTL3-overexpressing or control tumors (Fig. 8c). The tumor inhibition rate of everolimus in METTL3-high tumors was significantly higher than that in control tumors (89.92% vs. 73.26%, *P* = 0.0465, Fig. 8c–e), suggesting that METTL3-high tumors could be inhibited by everolimus more effectively.

## Discussion

Due to its heterogeneity and specificity, gastric cancer has been gradually shown to lack common driven mutations or CNVs that are commonly seen in lung, breast, or colorectal cancer. Consequently, it is difficult to copy the success of the translational therapies based on genetic alterations in certain types of cancers, such as the EGFR or ALK-driven non-small cell lung cancer. Increasing scholars believe that cancer is an epigenetic disease, and disrupted and unstable epigenomes exist widely among different types of tumors with heterogeneity and therapeutic resistance^15,16^. m^6^A is the most abundant internal modification in eukaryotic RNA and may represent a critical mechanism for regulating malignant behaviors of tumors^17^. Our data show that the m^6^A level was upregulated in gastric cancers and suppressing m^6^A by METTL3-knockdown hindered tumor growth and metastasis in vitro and in vivo. Based on these discoveries, we believe that abnormal m^6^A modification is an important epigenetic feature of gastric cancer and a potential therapeutic target for further study.

As the predominant “writer” of m^6^A, METTL3 is dysregulated and plays dual roles in cancers, coupled with different substrates and cell types^18^. It directly regulates transcription, translation, and RNA maturation of a broad range of oncogene and onco-suppressors, many of which are undiscovered, and the role of METTL3 cancers depends on orchestration of multiple effects^18^. In non-tumor cells, METTL3 methylated primary miRNAs, such as pri-let-7e and pri-miR221, and facilitated their maturation^19^. Recent studies reported that METTL3 accelerated the maturation of pri-miR221/222^20^ and pri-miR-25^21^ in bladder and pancreatic cancers. So far, there has been no study reported the regulating function of METTL3 on ncRNA in gastric cancer. As we found, one of the most prominent substrates of METTL3 is a non-coding primary miRNA, pri-miR-17-92, a 6-tandem stem hairpin-containing polycistronic transcript of the gene *MIR17HG* that encodes a miRNA cluster composed of 6 onco-miRNAs^22^. Through m^6^A modification, METTL3 facilitated pri-miR-17-92 to bind to DGCR8, which recognizes the stem-flanking junctions and orients DROSHA to cleave primary miRNA into precursor miRNA^23,24^. Our study revealed that the over-m^6^A modification on pri-miR-17-92, instead of its overexpression, caused upregulation of the miR-17-92 cluster and gastric cancer progression. To our knowledge, this is the first report of METTL3-mediated-m^6^A-dependent maturation of ncRNA and its downstream signal pathway and biological effect in gastric cancer. Most importantly, we mapped pri-miR-17-92 and found its A879 was the dominant site responsible for the m^6^A-mediated pri-miR-17-92 maturation because A879C mutation significantly abolished the METTL3-mediated m^6^A modification, DGCR8 binding, and miR-17-92 cluster formation. Therefore, the pri-miR-17-92 A879 represented the key mediator of the m^6^A-mediated pri-miR-17-92 maturation and was likely a highly precise target for intervention as an epigenetically modifiable position.

The regulation of the miR-17-92 cluster remains largely unclear, other than that c-Myc directly binds to *MIR17HG* and promotes pri-miR-17-92 transcription^25^. Our findings uncovered another layer of regulation of this miRNA cluster by m^6^A-mediated processing. Widely accepted as an onco-miRNA^26^, the miR-17-92 cluster targets many onco-suppressing genes, such as SMAD2 and SMAD4^27^, p21^28^, and TRAF3^29^. In this study, we found that TMEM127 and PTEN were part of the targets. Furthermore, forced expression of the miR-17-92 cluster counteracted the effects of METTL3-knockdown in PTEN/TMEM127 regulation, cell proliferation, and metastasis. TMEM127 is a negative regulator of the mTOR pathway and a tumor suppressor located on chromosome 2q11^30^. So far, the function of TMEM127 in gastric cancer and its association with the miR-17-92 cluster have not been reported. In this study, we did not observe frequent mutations or deletions of TMEM127. Instead, TMEM127, along with PTEN and the mTOR pathway, was efficiently regulated by the miR-17-92 cluster as its target. Therefore, we suspect that METTL3/miR-17-92 cluster activates mTOR pathways by targeting PTEN and TMEM127 in gastric cancer progression. Of note, several other signaling pathways, including p53, TGF-beta, and MAPK signaling pathways, were probably also involved, which are of value for further investigation in future studies.

Currently, drugs targeting epigenetic changes, such as DNA methyltransferase inhibitors decitabine, have become standard treatments in hematological malignancies^31^. Up to now, the small molecule inhibitor against METTL3 is not available. Based on the above discovery, the mTOR inhibitor everolimus was chosen to interfere with METTL3/miR-17-92 cluster/TMEM127 or PTEN/mTOR signaling pathway in gastric cancer. We found that everolimus indeed reversed the METTL3-induced proliferation in a dose-dependent manner. This effect was remarkably pronounced when METTL3 was highly expressed. The high sensitivity to everolimus in METTL3-high cells could be because the mTOR pathway was greatly activated by METTL3 in these cells. These data confirmed that the oncogenic role of METTL3 relies on mTOR activation in another aspect. Everolimus has shown promising efficacy in patients with previously treated advanced gastric cancer in a phase II study^32^. However, phase III studies^33–35^ failed to repeat the positive results, except for that the progression-free survival was significantly prolonged. Our findings provided evidence that the METTL3 level might be a potential predictor for treatment efficacy of everolimus in gastric cancer and may help screen possible advantageous subgroups out from the previous failed clinical trials with everolimus-treated gastric cancer. These inferences are yet to be further studied and confirmed in clinical trials.

In conclusion, METTL3/m^6^A promotes gastric cancer growth and metastasis by facilitating pri-miR-17-92 processing into the oncogenic miRNA cluster and activating the AKT/mTOR pathway by targeting PTEN and TMEM127, which could be targeted by everolimus (Fig. 8f). These findings provide us with a novel insight into the role of METTL3 in the regulation of cancer development and a theoretical rationale for use of everolimus in the treatment of m^6^A/METTL3-high gastric cancer.

## Supplementary information

Supplementary Figure Legends

Supplementary Figure 1

## Data Availability

Expression data of mRNA and miRNA in TCGA-STAD were downloaded from the GDC data portal (https://portal.gdc.cancer.gov). Survival data from GEO databases, including GSE14210, GSE15459, GSE22377, GSE29272, GSE38749, GSE51105, and GSE62254, were extracted from Kaplan–Meier Plotter (http://kmplot.com). Genomic alternation data were extracted from cBioPortal (http://www.cbioportal.org). All the other data and materials are available from the corresponding author upon reasonable request.
