# Supplementary material for: N6-methyladenosine-dependent pri-miR-17-92 maturation suppresses PTEN/TMEM127 and promotes sensitivity to everolimus in gastric cancer
Source: Cell Death Dis. 2020 Oct 9;11(10):836. doi: 10.1038/s41419-020-03049-w (PMC7547657; doi:10.1038/s41419-020-03049-w)
Supplement: Supplementary file 1 — Supplementary Figure Legends [file 41419_2020_3049_MOESM1_ESM.docx]

**Supplementary Figure Legends**

**Supplementary Fig. 1** Confirmation of the phenotype specificity of METTL3 knockdown by an alternative shRNA. **a** METTL3 expression in HGC-27 cells transfected with lentivirus encoding an shRNA against METTL3 (shM3-2) and control (shCTL). **b** Percentage of m^6^A content in total RNA in HGC-27 transfected with shM3-2 and shCTL. **c** Proliferation curves of HGC-27 cells transfected with shM3-2 and shCTL. **d** Colony-formation assays of HGC-27 cells transfected with shM3-2 and shCTL. **e** Wound-healing assays of HGC-27 cells transfected with shM3-2 and shCTL. **f** Migration assays of HGC-27 cells transfected with shM3-2 and shCTL. **g** Invasion assays of HGC-27 cells transfected with shM3-2 and shCTL. Representative pictures are shown on the left, and quantification on the right in **d-g**. Data are presented as mean ± SD. **P* < 0.05; ***P* < 0.01; ****P* < 0.001.
